# Organizational commitments to equality change how people view women’s and men’s professional success

**DOI:** 10.1038/s41598-024-56829-1

**Published:** 2024-03-31

**Authors:** Kristin Kelley, Lena Hipp, Paula Protsch

**Affiliations:** 1 American Institutes for Research, Arlington, VA, USA; 2 WZB Berlin Social Science Center, Berlin, Germany; 3 University of Potsdam, Potsdam, Germany; 4 University of Cologne, Cologne, Germany; 5 BIBB Federal Institute for Vocational Education and Training, Bonn, Germany

**Keywords:** Psychology, Human behaviour

## Abstract

To address women’s underrepresentation in high-status positions, many organizations have committed to gender equality. But is women’s professional success viewed less positively when organizations commit to women’s advancement? Do equality commitments have positive effects on evaluations of successful men? We fielded a survey experiment with a national probability sample in Germany (N = 3229) that varied employees’ gender and their organization’s commitment to equality. Respondents read about a recently promoted employee and rated how decisive of a role they thought intelligence and effort played in getting the employee promoted from 1 “Not at all decisive” to 7 “Very decisive” and the fairness of the promotion from 1 “Very unfair” to 7 “Very fair.” When organizations committed to women’s advancement rather than uniform performance standards, people believed intelligence and effort were less decisive in women’s promotions, but that intelligence was more decisive in men’s promotions. People viewed women’s promotions as least fair and men’s as most fair in organizations committed to women’s advancement. However, women’s promotions were still viewed more positively than men’s in all conditions and on all outcomes, suggesting people believed that organizations had double standards for success that required women to be smarter and work harder to be promoted, especially in organizations that did not make equality commitments.

## Introduction

During the U.S. presidential election campaign in 2020, Vice President Joe Biden promised to select a woman as his vice-presidential running mate. Proponents of this commitment claimed it was an important move toward rectifying women’s underrepresentation in leadership positions, while opponents argued that the announcement would lead people to devalue the not-yet-selected woman’s credentials^1^. Similarly, organizational commitments to gender equality may lead employees and the broader public to question whether the promoted employees are deserving or qualified. In fact, prior research on the effects of diversity values and affirmative action policies suggests that a woman’s performance will be scrutinized when her organization commits to women’s advancement^2^.

Experimental studies have found that people view women beneficiaries of affirmative action as less competent than nonbeneficiaries and believe affirmative action procedures are unfair^3–7^. Affirmative action had stigmatizing qualities, even when it was unclear whether potential recipients actually benefitted from the policy^4^. When women employees were equally qualified to men, participants’ negative evaluations of women who were hired or promoted under affirmative action or diversity initiatives were weakened but not completely eradicated^3^. Unless there was unambiguous information about the overall competence of the selected woman leader (i.e., a statement that she had performed amongst the top 5% of employees), participants viewed women beneficiaries as less competent and procedures as less fair when women were hired through programs designed to increase diversity^5^. However, unambiguous performance capacity is rarely available to external evaluators^5^.

There are several research gaps that need to be filled to gain a comprehensive understanding of whether and how efforts to foster women’s professional advancement impact evaluations of employees’ promotions. First, several literature reviews revealed that much of the research on perceptions of affirmative action beneficiaries was conducted on small, nonprobability samples in the 1990s and early 2000s^2–7^. It is unclear whether findings from these studies conducted with student samples can be generalized to the broader public. Additionally, recent studies on gender stereotypes have found that the proportion of people that view women and men as equally competent has increased over time^8,9^, suggesting that evaluations of women promoted under diversity initiatives may have also changed.

Second, initiatives to increase women’s advancement today are not just referred to as affirmative-action programs^10^. For example, organizations may state that they are committed to “women’s advancement”, “equal opportunities”, or “diversity”, all of which may be viewed either as statements on nondiscrimination and the importance of merit or as signals that women are given preferential treatment. It is unclear how these subtler, more ambiguous statements influence how people view the success of potential beneficiaries and the fairness of their promotions. In the current study, we randomly assigned respondents to read about one of three organizational types. Our reference condition was an organization that values performance and uniform assessment standards and states that the most qualified employees are selected for leadership positions. There is no reference to gender or equality values. The next organization committed to women’s advancement. This common organizational signal^11^ may lead respondents to believe that the organization gives preferential treatment toward women. The final organization committed to equal opportunities. Equal opportunity may signal preferential treatment toward woman to a lesser degree, because the focus is on equality rather than specifically on women.

Third, prior research has compared women in preferential treatment conditions to women and men in non-preferential treatment conditions. However, research has not examined how perceived preferential treatment of women within one organization may affect evaluations of men within that organization. That is, it is unknown whether organizational commitments to gender equality change perceptions of successful male employees. The public may give more favorable evaluations of men who are successful in equality-oriented organizations if they believe equality initiatives make it harder for men to be promoted. With this study, we seek to close these research gaps.

Drawing on data from a survey experiment conducted on a national probability sample in Germany (N = 3229), we examine how perceptions of recently promoted women and men differ depending on whether their organizations were described as committed to performance and uniform assessment standards (hereafter, uniform performance standards), women’s advancement, or equal opportunities. More specifically, we ask: Do people give lower attributions to intelligence and effort and view promotions as less fair when women are promoted in organizations committed to women’s advancement or equal opportunities compared to uniform performance standards? Do organizational equality commitments enhance attributions of men’s success and the perceived fairness of men’s promotions?

### Success attributions

To understand how gender and organizational commitments to gender equality may influence the perceived reasons for employees’ promotions, we draw on insights from attribution theory^12,13^. Specifically, we consider how the public attributes employees’ promotions to intelligence and effort. Intelligence—sometimes referred to as ability—and effort—sometimes referred to as hard work—are viewed as the most justifiable explanations for professional success in Western societies^14,15^. Intelligence and effort are the two core components of professional/work-related competence^15,16^. While previous work combined measures of perceived intelligence and effort to capture competence and status^15–17^, it is also important to consider the two measures separately because intelligence and effort may not always align. For example, stereotypes that men’s success is due to greater intelligence and that women’s success is due to effort remain pervasive in some contexts^18,19^.

According to attribution theory, people act as “lay psychologists” and attempt to explain why certain events occur^12^. People attribute events to internal factors when they believe the event is a result of an individual’s core character or external factors when they believe that the event was influenced by the situation or other outside forces^12,13^. Studies on gender inequalities have used these insights to understand whether women and men are evaluated differently for the same behaviors or achievements^20–22^. For example, a recent study found that respondents were more likely to attribute men’s knowledge to ability^23^.

Attribution theory suggests that people are more likely to form external attributions when events run counter to expectations^13,24^. Historically, prevalent stereotypes suggest that women are not well-suited to high-status occupations and leadership roles and therefore people have looked for “an attributional ‘out’ for explaining away the existence of women at high levels”^25^. For example, if a woman is promoted in an organization committed to gender equality, people may presume that she received extra support and attribute her success to that support rather than to intelligence and effort^14,15^. In other words, people who do not believe women can be successful on their own merits may attribute women’s professional success to affirmative action programs or diversity initiatives, which they presume give preferential treatment to less qualified women over more qualified men. Prior research has found evidence of these effects^3–7^. If people perceive that diversity initiatives are responsible for women’s success, they will be less likely to attribute women’s success to factors such as intelligence and effort^15^.

Additionally, the public may believe that equality initiatives make it more challenging for nonbeneficiaries to be successful. When people are professionally successful in spite of perceived constraints, they are given higher attributions to intelligence and effort^26^. Therefore, equality initiatives may make nonbeneficiaries appear very competent when they are promoted or hired^27^. For example, people may view women’s advancement commitments as a barrier to men’s success. Therefore, if men are promoted in organizations with gender equality initiatives, people may believe men had to work harder (i.e., exert more effort) and be smarter (i.e., more intelligent) than they would have in an organization that did not externally commit to gender equity.

In summary, organizational commitments to women’s advancement may backfire through two mechanisms—they may lead people to lower their attributions to women’s intelligence and effort but raise their attributions to men’s intelligence and effort. Specifically, we predict that people will believe intelligence plays a less decisive role in women’s promotions (H1) but a more decisive role in men’s promotions (H2) when organizations commit to women’s advancement rather than uniform performance standards. Likewise, we predict that people will believe effort plays a less decisive role in women’s promotions (H3) but a more decisive role in men’s promotions (H4) when organizations commit to women’s advancement rather than uniform performance standards.

### Fairness evaluations

Organizational equality commitments might also impact the perceived fairness of employees’ promotions, given that “cognitive processing about causes or attributions underlies justice assessments”^26^. According to *distributive justice theory*, the fairness of a situation may be judged based on equality, equity, or needs-based principles^28^. Within societies that value meritocracy, like the United States and Germany, people typically rely on the equity principle to determine fairness^29^. The equity principle states that competitions (e.g., promotion decisions) should be judged based on individuals’ inputs, and that employees with the highest intelligence and effort, should be awarded^26^. Scholars have applied this framework to examine various outcomes, including poverty attributions and perceived fairness^30^. According to *procedural justice theory*, people consider organizational practices, policies, and procedures when making fairness evaluations^26,31,32^. Specifically, people rely on procedural information to manage uncertainty and determine fairness in ambiguous situations, like when evaluators have little information regarding the specifics of the hiring process. This robust finding is called the “fair process effect”^33,34^.

Because equality commitments may suggest that employees’ sociodemographic characteristics, such as gender, and not just intelligence and effort, are considered in decision-making, we predict that *people will believe women’s promotions are less fair when organizations are committed to women’s advancement rather than uniform performance standards (H5).* By the same logic, people may believe men’s promotions are fairer when organizations are committed to women’s advancement.

## Results

To test our hypotheses, we analyzed data from a preregistered^35^ vignette-based survey experiment on a national probability sample in Germany (N = 3229). We randomly varied whether a recently promoted employee was a woman or a man. The hypothetical employees were in their early 30s and lawyers in a large company. We also varied whether the employee’s organization was committed to women’s advancement, equal opportunities, or uniform performance standards. After reading the vignette, respondents rated how decisive they thought “intelligence” and “effort” were in the employee’s promotion from 1 (*not at all decisive*) to 7 (*very decisive*). Respondents also rated the perceived fairness of the promotion from 1 (*very unfair*) to 7 (*very fair*).

**Figure 1:**
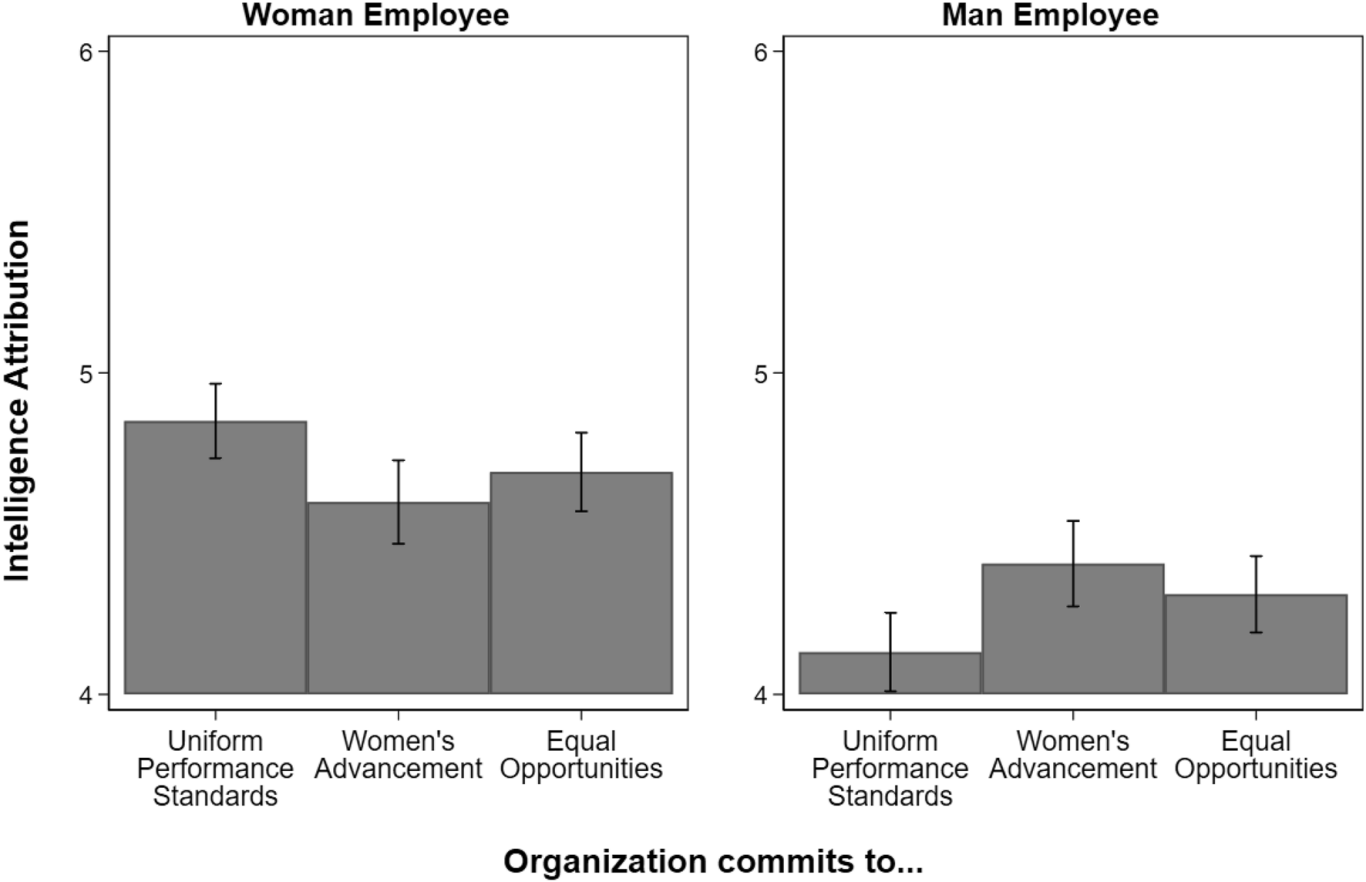
Degree to which promotions were attributed to intelligence from 1 (lowest) to 7 (highest), by employee gender and organizational type. N = 3229 (1613 evaluations of women employees; 1616 evaluations of men employees). Figure displays predicted values with 95% confidence intervals. Organizations committed to uniform performance standards is the reference category. People believed intelligence played a less decisive role in women’s promotions when they were promoted in organizations committed to women’s advancement (β =  − 0.25, p < 0.01, Table S3) and equal opportunities (β =  − 0.16, p < 0.1, Table S3). People believed intelligence played a more decisive role in men’s promotions when they were promoted in organizations committed to women’s advancement (β = 0.28, p < 0.01, Table S3) and equal opportunities (β = 0.18, p < 0.05, Table S3).

**Figure 2:**
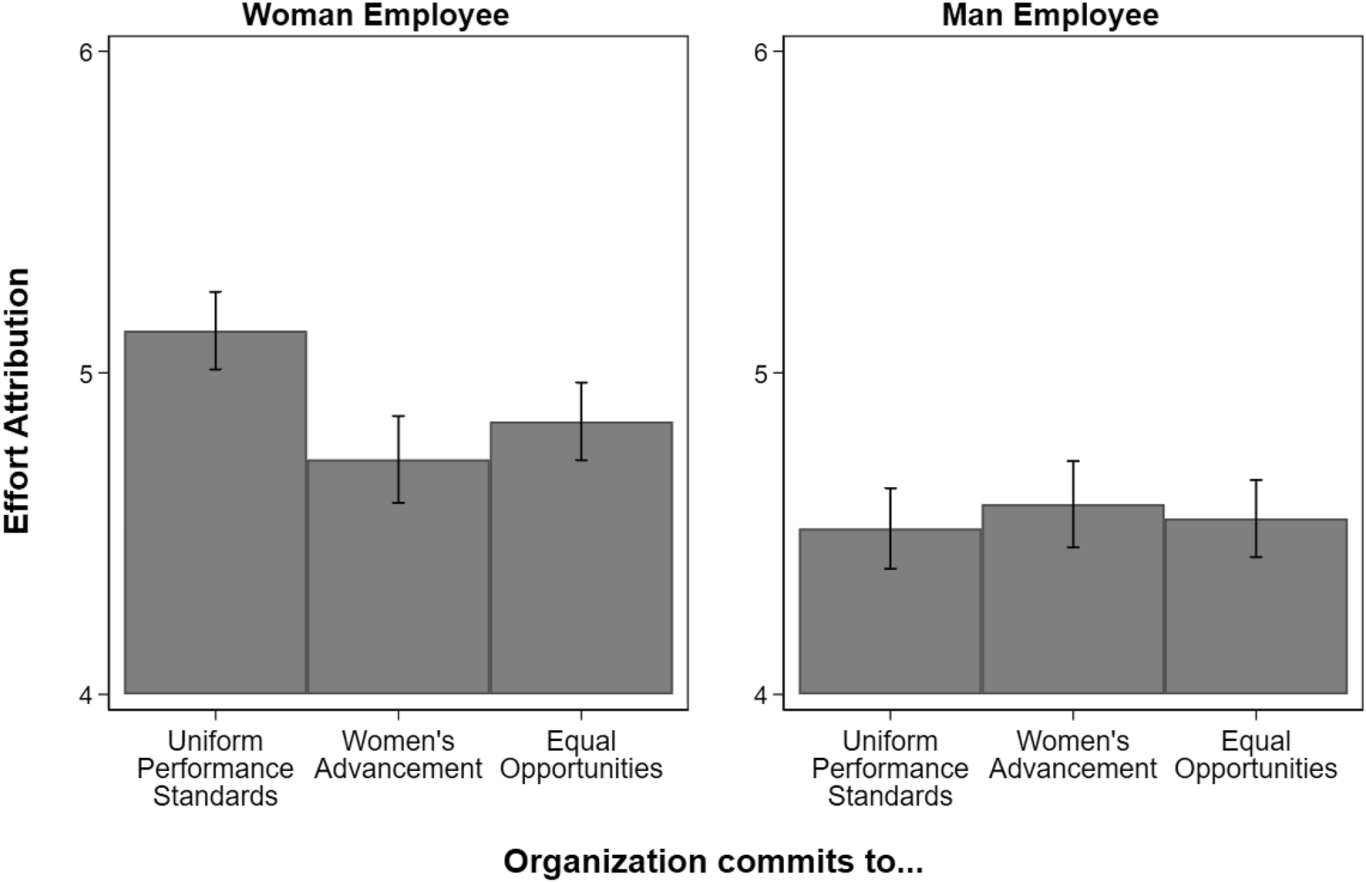
Degree to which promotions were attributed to effort from 1 (lowest) to 7 (highest), by employee gender and organizational type. N = 3229 (1613 evaluations of women employees; 1616 evaluations of men employees). Figure displays predicted values with 95 percent confidence intervals. Organizations committed to uniform performance standards is the reference category. People believed effort played a less decisive role in women’s promotions when they were promoted in organizations committed to women’s advancement (β =  − 0.40, p < 0.001, Table S3) or equal opportunities (β =  − 0.28, p < 0.01, Table S3). People did not view the role of effort in men’s promotions differently across organizational types.

**Figure 3:**
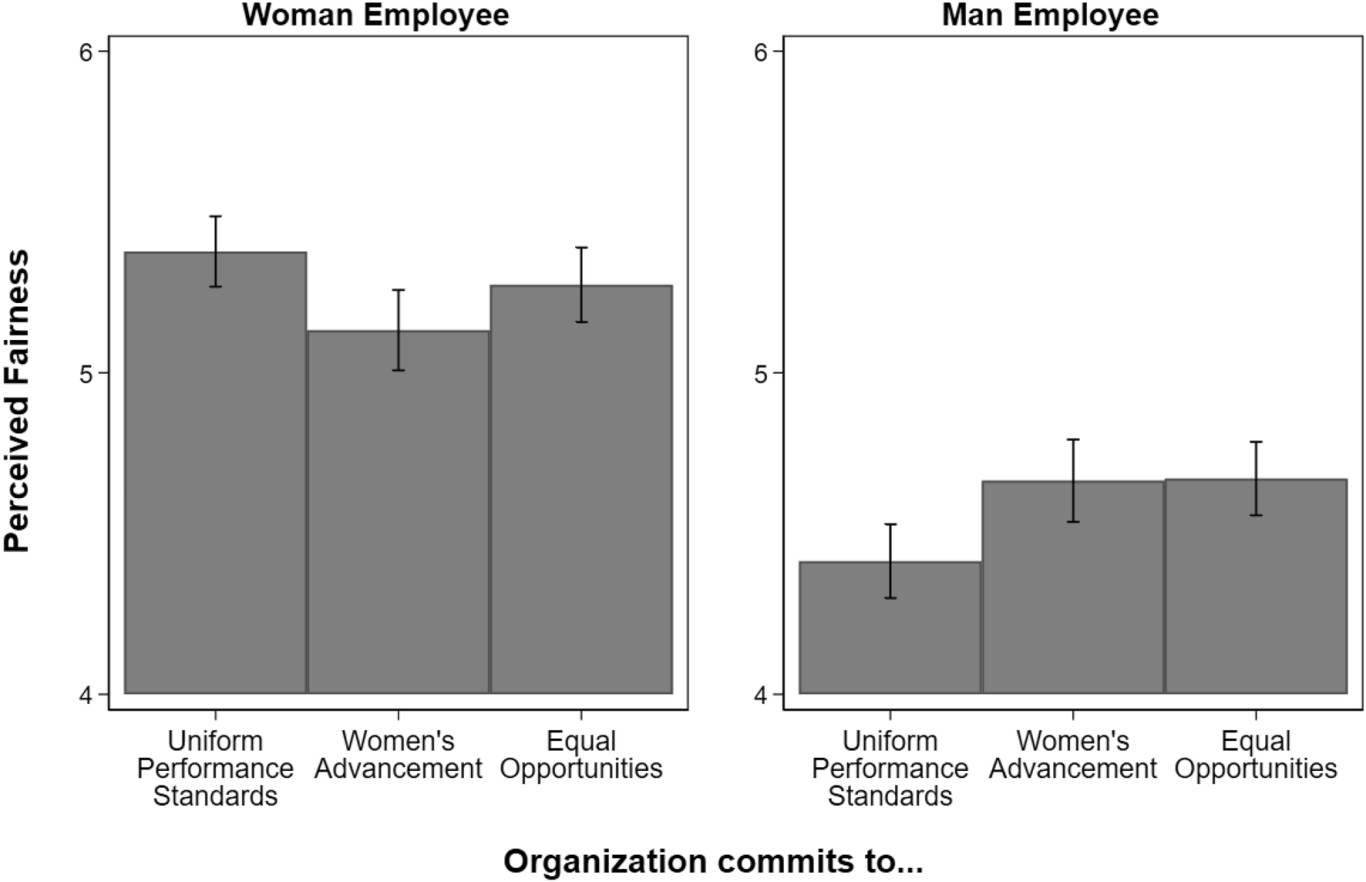
Perceived fairness of employees’ promotions from 1 (lowest) to 7 (highest), by employee gender and organizational type. N = 3229 (1613 evaluations of women employees; 1616 evaluations of men employees). Figure displays predicted values with 95 percent confidence intervals. Organizations committed to uniform performance standards is the reference category. People believed women’s promotions were less fair when they were promoted in organizations committed to women’s advancement (β =  − 0.25, p < 0.01, Table S3). People did not view the fairness of women’s promotions differently in organizations committed to equal opportunities. People believed men’s promotions were fairer when they were promoted in organizations committed to women’s advancement or equal opportunities (β = 0.25, and β = 0.26, both p < 0.01, Table S3).

Our main analyses test the impact of organizational commitments by employee gender. Therefore, we estimated gender-stratified linear regression models with respondent-level covariates (see Table S3). Figures 1, 2 and 3 display predicted values with 95% confidence intervals based on the gender-stratified models. As a supplement to our main analyses, we also estimated and reported the impact of gender across organizational commitments. Specifically, we report contrasts of the marginal linear predictions estimated based on regressions with an interaction between organizational commitment and employee gender (see Tables S4, S5). There are more details in the “Materials and methods” section.

### Intelligence attributions

Figure 1 shows that when women were promoted in organizations committed to women’s advancement, people believed intelligence played a less decisive role than when they were promoted in organizations committed to uniform performance standards (β =  − 0.25, p < 0.01, Table S3), supporting our first hypothesis. We observed a somewhat smaller and statistically insignificant decrease in intelligence attributions when women were promoted in organizations committed to equal opportunities (β =  − 0.16, p < 0.1, Table S3).

In contrast to ratings of women’s promotions, when men were promoted in organizations committed to women’s advancement, people believed intelligence played a more decisive role than when men were promoted in organizations committed to uniform performance standards (β = 0.28, p < 0.01, Table S3), supporting our second hypothesis. People also gave higher intelligence attributions to men promoted in equal opportunity organizations than men in organizations committed to uniform performance standards (β = 0.18, p < 0.05, Table S3).

Despite the decreased attributions to women’s intelligence and the increased attributions to men’s intelligence, people rated attributions to women’s intelligence higher than men’s in each organizational type. The largest gender gap in predicted values was in organizations committed to uniform performance standards (0.70, p < 0.001, Table S5), followed by organizations committed to equal opportunities (0.37, p < 0.001, Table S5), and the smallest was in organizations committed to women’s advancement (0.17, p < 0.1, Table S5).

### Effort attributions

Figure 2 shows that the pattern of effects for effort attributions is like that for intelligence attributions. When women were promoted in organizations committed to women’s advancement, people believed effort played a less decisive role in their promotions than in organizations committed to uniform performance standards (β =  − 0.40, p < 0.001, Table S3), supporting our third hypothesis. This decrease in attributions to women’s effort also occurred when organizations committed to equal opportunities (β =  − 0.28, p < 0.01, Table S3). The negative effect of a commitment to women’s advancement, however, was larger than the effect of a commitment to equal opportunities. Contrary to our fourth hypothesis, we did not find that people gave higher effort attributions to men promoted in organizations committed to women’s advancement (or equal opportunities).

As with the intelligence attributions, people rated attributions to women’s effort higher than men’s in each type of organization. Again, the largest gender gap in predicted values was in organizations committed to uniform performance standards (0.60, p < 0.001, Table S5), followed by organizations committed to equal opportunities (0.29, p < 0.001, Table S5), and the smallest was in organizations committed to women’s advancement (0.13, n.s., Table S5).

### Fairness evaluations

Figure 3 displays the results for fairness evaluations. People perceived women’s promotions as less fair when they worked in organizations committed to women’s advancement than in organizations committed to uniform performance standards (β =  − 0.25, p < 0.01, Table S3), supporting our fifth hypothesis. There was no statistically significant difference in the perceived fairness of women’s promotions between organizations that were committed to equal opportunities and uniform performance standards.

Again, in contrast to evaluations of women’s promotions, men’s promotions were viewed as fairer in organizations committed to women’s advancement and equal opportunities than in organizations committed to uniform performance standards (β = 0.25 and β = 0.26, both p < 0.01, Table S3).

As is the case for the attribution outcomes, people viewed women’s promotions as fairer than men’s in each type of organization. The largest gender gap in predicted values was in organizations committed to uniform performance standards (0.93, p < 0.001, Table S5), followed by organizations committed to equal opportunities (0.58, p < 0.001, Table S5), and the smallest was in organizations committed to women’s advancement (0.45, p < 0.001, Table S5).

## Discussion

The current study shows that when organizations commit to women’s advancement, people believe that intelligence and effort play a less decisive role in women’s promotions and that women’s promotions are less fair. In contrast, people believe intelligence plays a more decisive role in men’s promotions and view men’s promotions as fairer when organizations commit to women’s advancement rather than uniform performance standards. Our findings regarding the effects of equality commitments on evaluations of women show that findings from earlier studies based on nonprobability samples are generalizable^3–7,29^. Additionally, our study generated novel findings regarding how succeeding in organizations that commit to equality positively affects evaluations of men’s promotions. While previous research focused on how perceptions of women’s professional success are affected by organizational commitments to equality, their impact on evaluations of men’s success was unclear.

What mechanisms underlie our findings? Our results suggest that organizational commitments provide clues to how organizations make promotion decisions. That is, evaluators may infer the criteria that organizational decision makers use in promotion processes from organizations’ value statements. Organizational commitments to women’s advancement may signal that women received some form of extra help to get promoted, or that they were evaluated by more lenient standards. This explanation supports our finding that the public believes that intelligence and effort play less of a role in women’s promotions in organizations that commit to women’s advancement. Our results also suggest that evaluators assumed that men were evaluated by more rigorous standards than women when organizations committed to women’s advancement or equal opportunities. In turn, they gave higher ratings to men’s intelligence and effort when men worked for organizations committed to women’s advancement than when men worked for organizations committed to uniform performance standards.

Yet, the findings also showed that people believe intelligence and effort play a more decisive role in women’s promotions and that women’s promotions are fairer than men’s in each organizational type. These gender differences were largest when organizations committed to uniform performance standards and smallest when organizations committed to women’s advancement. Again, these results suggest that the public infers organizations’ decision-making criteria based on organizational commitments, and these inferences shape whether evaluators perceive that intelligence and effort played a large role in promotions. According to justice theory, people give greater attributions to internal factors and perceive greater justice for people who are successful despite facing constraints^26^. Our results suggest that the public believed that organizational decision makers relied on sexist double standards when making promotion decisions^36^. Specifically, they may have thought that women were more likely to have their job performances scrutinized and be held to higher evaluation standards. This explains why the public rated women higher than men on attributions to intelligence and effort and rated women’s promotions as fairer than men’s promotions. In summary, our findings suggest that organizational commitments to uniform performance standards lead the public to think that decision-makers are sexist, and thus, that women must be smarter and work harder than men to be promoted.

The largest gender gaps in evaluations are in organizations committed to uniform performance standards. People may believe these organizations have particularly sexist decision-making processes or structures that create barriers for women. Terms like “performance” and “uniform assessment standards” may signal that organizations value people who are available 24/7 and who meet the “ideal worker” norm^37^. Given that the burdens of caregiving and housework still primarily fall on women, people may believe that it is more difficult for women to succeed in performance-oriented organizations. Moreover, people may expect biases against women to persist, even when organizations commit to seemingly meritocratic principles. This possibility is in line with prior research that found managers were more biased when evaluating employees with meritocratic standards than when asked to evaluate them based on neutral criteria that did not explicitly mention meritocratic values^38,39^.

People evaluated employees promoted in organizations committed to equal opportunities and women’s advancement similarly. However, the differences between committing to equal opportunities versus uniform performance standards are smaller than the differences between committing to women’s advancement versus uniform performance standards. This is consistent with prior research, which suggested that any type of equality commitment affects how individuals are viewed, but that the impact is stronger if initiatives give more weight to demographic characteristics^3,29,40,41^. People may have believed that when organizations committed to women’s advancement, decision-makers gave more weight to gender in decision-making processes than when organizations committed to equal opportunities. The results suggest that people believed that employees’ gender was considered in the promotion process in organizations committed to women’s advancement and those committed to equal opportunities, but that they believed gender played a more salient role when organizations explicitly mentioned women.

The findings of our research have important implications for both organizational and political decision-making. Prior research claimed that organizations should exercise caution when seeking to actively increase their demographic diversity^7^. Our findings, however, suggest that the concern that equality commitments backfire through stigmatization of presumed beneficiaries may be unwarranted. We found that evaluations of women’s and men’s promotions were most similar when organizations committed to women’s advancement, suggesting people believe these organizations are successful at eradicating most of the sexism in the promotion process. Therefore, our findings support the implementation of organizational commitments to equality to the extent that these organizations are implementing policies, programs, and practices that actually help women advance and support them on the job, such as social accountability systems and work-life programs^42^. If organizations are outwardly committed to women’s advancement but using different metrics or processes to evaluate women and men, this could be particularly harmful for women’s advancement because the inequalities will be hidden behind seemingly egalitarian values. After all, gender bias is perpetuated most by those who do not believe inequalities exist^10,43^. Special consideration should be given to how to frame equality commitments that gain the support of those who are most skeptical.

To extend our collective knowledge on the impacts of equality and diversity initiatives, future research should consider how the effects identified in the current research generalize to other contexts and outcomes. For example, do our results generalize to occupations that are lower status or that have different gender ratios? Additionally, although Germany and the United States share many characteristics, there could be some country-level differences. Scholars may also consider whether alternative framings of equality commitments, such as a “commitment to diversity”, have similar impacts on success attributions and fairness evaluations. In light of the finding that white able-bodied heterosexual men experience better career opportunities than members of various other intersectional groups^44^, future research should consider whether evaluations differ across employees based on their particular constellation of identities. Future studies could also examine the impact of equality commitments on other outcomes, such as hiring and pay raises.

## Materials and methods

### Data

This experiment was implemented in a survey on work values and attitudes in Germany in early 2023. Using a national probability sampling scheme, 6000 respondents that were 23–65 years old were invited via mail to participate in the online study. Potential respondents were incentivized with a €10 post-hoc payment for study participation. The experimental protocol received ethical approval from the WZB Berlin Social Science Center Ethics Committee prior to collecting the data and informed consent was obtained from all participants. All research was performed in accordance with the relevant guidelines. Following our preregistered analysis plan^35^, we excluded respondents who failed the manipulation check (967 of 4211 respondents). We also dropped data from 15 respondents who were missing data on variables included in the analysis, which led to an analytic sample of 3229 respondents. Older respondents and respondents with higher education were more likely to participate, which is common for survey research^45,46^. See Table S1 for the demographic characteristics of the initial and analytic samples.

### Context

Germany shares many structural and economic features with other rich democracies, making the findings relevant for many countries. Despite major political advances to promote gender equality, such as the introduction of a Swedish-style parental leave scheme in the early 2000s and a liberalization of public opinion regarding mothers’ employment^47^, Germany is still characterized by a traditional gender division of paid and unpaid work^48,49^, as well as high levels of income inequality between women and men^50^. While women’s labor force participation has consistently risen in recent decades, it still lags 8 percentage points behind men’s^51^, and women are still underrepresented in leadership positions. The proportion of women in management positions in Germany is 29%, well below the European average of 35%^51^. A broad array of policy and organizational level measures have been established to close this gender authority gap^52^. For example, in Germany public firms have been mandated to implement policies to foster equality since 1994 and the majority of private sector employees also work in firms with organizational policies that promote gender equality^11^.

### Experimental design

Relying on a between-subjects design, we randomly assigned respondents to read one vignette that described an employee who was recently promoted. We varied three dimensions of the vignette. First, we varied the employee’s gender by indicating her/his first name; we selected a typically female and a typically male name from common first names in Germany in the year 1990 (when employees in their early 30s would have been born)^53^. Second, we varied whether the employee’s current employer was committed to “performance and uniform assessment standards”, “women’s advancement”, or “equal opportunities”. Our objective was to signal organizations’ general commitments rather than to specifically state that the employee did or did not benefit from an affirmative action program. This increases external validity because it is unlikely that people would know the exact details behind an employee’s promotion, but the organization’s general position on equality would be common knowledge^4,54^. Third, we varied whether the employee’s competitor in the promotion contest was a woman or man, which is indicated by the ending for the German word for competitor (“Bewerberin”/“Bewerber”). The competitor dimension is included in all models, but treated as a covariate rather than an independent variable. With this design, our findings should generalize to same-sex and different-sex promotion contests.

We held the following factors constant in the vignette: target employees’ marital and parental status, age, occupation, and work performance. All employees were described as married with two children, because most employees do have or will have children. We chose lawyer as the occupation because it is one of the few gender-balanced high-status occupations in Germany (52 percent of lawyers were women in 2022)^55^. We described employees’ work performance as “average to good” to ensure maximum variation across outcomes. A vignette with each variation of the manipulated dimensions is shown below:

[Julia/Tobias] Müller, early 30s, married, two children, has been working as a lawyer in a large company for several years and has recently been promoted. [Her/his] work was always rated average to good. [Her/his] employer commits to [performance and uniform assessment standards/women’s advancement/equal opportunities]. [She/he] succeeded in getting the promotion against a similarly qualified [woman/man] competitor.

### Dependent variables

After reading the vignette, respondents were asked “How decisive do you think the following factors were in getting [Julia/Tobias] promoted?” for both “intelligence” and “effort”. Answer options ranged from 1 (*not at all decisive*) to 7 (*very decisive*). They were also asked, “How fair do you think it is that [Julia/Tobias] was promoted?” from 1 (*very unfair*) to 7 (*very fair*). The order in which the questions were asked was randomized. See Table S2 for the means and standard deviations of the dependent variables for each of the six conditions.

### Statistical analysis

We relied on our preregistration to guide our analyses^35^. We estimated six OLS regression models (one for each outcome variable, stratified by employee gender). As our tests of OLS assumptions revealed that error terms were not homoscedastic across all models, we report results with robust standard errors. We included all manipulated dimensions in the models, as well as the following respondent-level characteristics: gender (woman, man, non-binary), relationship status (married or in a civil partnerships, other), children under 18 in the household (children, no children), age groups by decade (20s, 30s, 40s, 50s, 60s), region of upbringing (East Germany, West Germany, outside Germany), education (tertiary degree, no tertiary degree), and respondents’ subjective assessment of their financial situation (comfortable, getting by, hard or very hard to get by). We used an $\alpha$ level of 0.05 (two-tailed) for all statistical tests. In our pre-registration, we planned to estimate one-tailed statistical tests for our directed hypotheses, but we present the more conservative two-tailed tests. The results are displayed in Figs. 1, 2 and 3. See Table S3 for regression models.

We also examined how the impacts of being a woman employee changed across organizational commitments by estimating OLS regression models with interaction terms for employee gender and organizational commitments and robust standard errors. Following the regression models, we estimated the contrasts of marginal linear predictions of employee gender by the organizational commitment to facilitate the interpretation of the interaction effects. The contrasts of the marginal linear predictions show whether the gender gaps in success attributions and fairness evaluations significantly differ across types of organizational commitment. See Table S4 for the regression coefficients and Table S5 for the contrasts of marginal linear predictions.

### Sensitivity analyses

We tested that the results were robust to multiple model specifications. First, we estimated OLS regressions excluding respondent-level covariates and including respondents who failed the manipulation check (Tables S6–S8). Second, we estimated ordered logit regressions and regressions with box-cox transformed dependent variables as alternative ways to address the heteroscedasticity of the errors in some models (Tables S9, S10). Our results were robust to each of these alternative specifications.

### Supplementary Information

Supplementary Tables.

## Data Availability

The datasets used and/or analysed during the current study available from the corresponding author on reasonable request.
