# Supplementary material for: Organizational commitments to equality change how people view women’s and men’s professional success
Source: Sci Rep. 2024 Mar 31;14:7609. doi: 10.1038/s41598-024-56829-1 (PMC10982289; doi:10.1038/s41598-024-56829-1)
Supplement: Supplementary file 1 — Supplementary Tables. [file 41598_2024_56829_MOESM1_ESM.docx]

Supplementary Materials for

**Organizational commitments to equality change how people view women’s and men’s professional success**

**This PDF file includes:**

Tables S1 to S10

|  |  | |
| --- | --- | --- |
|  | Initial Sample^a^ | Analytic Sample^b^ |
| Age, by decade (range 23 – 65) |  |  |
| 20s | 0.041 | 0.044 |
| 30s | 0.114 | 0.114 |
| 40s | 0.191 | 0.185 |
| 50s | 0.350 | 0.354 |
| 60s | 0.304 | 0.303 |
| Gender identity |  |  |
| Women | 0.513 | 0.521 |
| Men | 0.485 | 0.478 |
| Non-binary | 0.002 | 0.002 |
| Marital status |  |  |
| Never married, widowed, divorced, or separated | 0.437 | 0.429 |
| Married or in a civil partnership | 0.562 | 0.571 |
| Missing data | 0.001 |  |
| Children under 18 in household |  |  |
| 0 children | 0.769 | 0.764 |
| 1 or more children | 0.231 | 0.236 |
| Region at birth |  |  |
| West Germany | 0.714 | 0.727 |
| East Germany | 0.259 | 0.249 |
| Outside Germany | 0.027 | 0.024 |
| Education |  |  |
| Less than tertiary degree | 0.552 | 0.528 |
| Tertiary degree or more | 0.448 | 0.472 |
| Subjective income assessment |  |  |
| Comfortable | 0.388 | 0.410 |
| Gets By | 0.465 | 0.457 |
| Hard to Get by | 0.147 | 0.134 |
| Current employment status |  |  |
| Working | 0.817 | 0.826 |
| Not Working | 0.183 | 0.174 |
| Total # Respondents | 4,211 | 3,229 |
| *Notes*. Original survey data collected by infas. Proportions are rounded to the nearest decimal point. ^a^Initial sample includes all respondents. ^b^Respondents in the analytic sample had no missing data and passed the manipulation check. | | |

Table S1. Respondent Demographic Characteristics.

|  | |  |  |  |  |
| --- | --- | --- | --- | --- | --- |
| Intelligence | | | Effort | Fairness | |
| Employee Gender | Organization commits to… | Mean (SD) | Mean  (SD) | Mean  (SD) | n |
| Women Employees | Uniform performance standards | 4.847  (1.409) | 5.132  (1.455) | 5.375  (1.325) | 555 |
|  | Women’s advancement | 4.603  (1.507) | 4.732  (1.580) | 5.134  (1.470) | 529 |
|  | Equal Opportunities | 4.690  (1.442) | 4.847  (1.427) | 5.274  (1.373) | 529 |
|  |  |  |  |  |  |
| Men Employees | Uniform performance standards | 4.127  (1.472) | 4.511  (1.512) | 4.410  (1.410) | 544 |
|  | Women’s advancement | 4.396  (1.545) | 4.579  (1.556) | 4.642  (1.521) | 503 |
|  | Equal Opportunities | 4.325  (1.437) | 4.562  (1.462) | 4.694  (1.407) | 569 |

Table S2. Means and Standard Deviations of Dependent Variables across Conditions. N= 3,229.

|  | Intelligence Attribution | | Effort Attribution | | Fairness | |
| --- | --- | --- | --- | --- | --- | --- |
|  | Women  Employees | Men  Employees | Women Employees | Men  Employees | Women Employees | Men Employees |
| Organization commits to... (ref. Uniform performance standards) |  |  |  |  |  |  |
| Women's advancement | -0.252^**^ | 0.275^**^ | -0.400^***^ | 0.076 | -0.245^**^ | 0.250^**^ |
|  | (0.089) | (0.092) | (0.092) | (0.094) | (0.085) | (0.088) |
| Equal opportunities | -0.158^+^ | 0.180^*^ | -0.282^**^ | 0.031 | -0.104 | 0.257^**^ |
|  | (0.086) | (0.087) | (0.087) | (0.089) | (0.082) | (0.083) |
| Competitor gender (ref. Man competitor) |  |  |  |  |  |  |
| Woman competitor | -0.145^*^ | -0.304^***^ | -0.028 | -0.297^***^ | -0.132^+^ | -0.647^***^ |
|  | (0.072) | (0.073) | (0.074) | (0.074) | (0.069) | (0.069) |
| Resp. gender (ref. Woman respondent) |  |  |  |  |  |  |
| Man respondent | -0.253^***^ | 0.253^***^ | -0.263^***^ | 0.252^***^ | -0.364^***^ | 0.300^***^ |
|  | (0.072) | (0.074) | (0.074) | (0.075) | (0.069) | (0.071) |
| Non-binary respondent | -0.472 | 0.303 | -0.332 | 1.440^*^ | 0.816^*^ | 0.646 |
|  | (0.432) | (0.316) | (0.216) | (0.673) | (0.354) | (0.582) |
| Age group in decades (ref. 20s) |  |  |  |  |  |  |
| 30s | -0.240 | -0.129 | -0.582^**^ | -0.331^+^ | -0.339^+^ | -0.434^*^ |
|  | (0.191) | (0.176) | (0.200) | (0.197) | (0.187) | (0.179) |
| 40s | -0.539^**^ | -0.179 | -0.652^***^ | -0.296 | -0.319^+^ | -0.447^**^ |
|  | (0.189) | (0.172) | (0.191) | (0.193) | (0.178) | (0.172) |
| 50s | -0.412^*^ | -0.186 | -0.624^***^ | -0.286 | -0.269 | -0.644^***^ |
|  | (0.173) | (0.163) | (0.175) | (0.184) | (0.167) | (0.160) |
| 60s | -0.233 | -0.186 | -0.503^**^ | -0.295 | -0.192 | -0.616^***^ |
|  | (0.177) | (0.169) | (0.181) | (0.189) | (0.171) | (0.164) |
| Resp. relationship status (ref. Single, divorced, or widowed) |  |  |  |  |  |  |
| Married or in civil partnership | 0.082 | 0.004 | 0.048 | 0.022 | -0.056 | 0.185^*^ |
|  | (0.083) | (0.080) | (0.085) | (0.082) | (0.080) | (0.076) |
| Resp. has children under age 18 in household (ref. No children) |  |  |  |  |  |  |
|  |  |  |  |  |  |  |
| Children under 18 in household | 0.045 | -0.027 | 0.059 | 0.012 | 0.025 | -0.075 |
|  | (0.099) | (0.093) | (0.102) | (0.097) | (0.095) | (0.095) |
| Resp. region of upbringing (ref. West Germany) |  |  |  |  |  |  |
| East Germany | 0.115 | -0.059 | 0.048 | -0.074 | 0.040 | -0.067 |
|  | (0.084) | (0.091) | (0.086) | (0.091) | (0.081) | (0.081) |
| Outside Germany | 0.102 | 0.361 | 0.143 | 0.546^*^ | -0.146 | 0.256 |
|  | (0.236) | (0.255) | (0.225) | (0.221) | (0.207) | (0.280) |
| Resp. education (ref. No tertiary degree) |  |  |  |  |  |  |
|  |  |  |  |  |  |  |
| Tertiary degree | -0.001 | -0.239^**^ | 0.005 | -0.335^***^ | 0.024 | -0.152^*^ |
|  | (0.076) | (0.081) | (0.077) | (0.081) | (0.073) | (0.077) |
| Resp. subjective income assessment (ref. can live comfortably) |  |  |  |  |  |  |
| Can get by | 0.028 | 0.185^*^ | 0.050 | 0.158^+^ | 0.134^+^ | -0.053 |
|  | (0.082) | (0.084) | (0.082) | (0.084) | (0.077) | (0.079) |
| Hard/very hard to get by | -0.012 | 0.178 | -0.003 | 0.023 | 0.017 | -0.033 |
|  | (0.131) | (0.129) | (0.130) | (0.126) | (0.123) | (0.125) |
| Resp. work status (ref. Working) |  |  |  |  |  |  |
| Not working | 0.013 | 0.217^*^ | 0.162 | 0.130 | 0.103 | 0.073 |
|  | (0.104) | (0.109) | (0.107) | (0.113) | (0.095) | (0.100) |
| Constant | 5.289^***^ | 4.332^***^ | 5.725^***^ | 4.898^***^ | 5.798^***^ | 5.187^***^ |
|  | (0.190) | (0.174) | (0.191) | (0.196) | (0.183) | (0.174) |
| *N* | 1613 | 1616 | 1613 | 1616 | 1613 | 1616 |
| *R*^2^ | 0.024 | 0.039 | 0.030 | 0.037 | 0.033 | 0.080 |
| adj. *R*^2^ | 0.013 | 0.029 | 0.020 | 0.027 | 0.022 | 0.070 |

Table S3. Linear Regression Estimates for Effects of Organizational Commitments by Employee Gender. + p < 0.10, * p < 0.05, ** p < 0.01, *** p < 0.001; Two-tailed tests. Robust standard errors in parentheses. Only 5 respondents identified as non-binary, so we caution readers not to interpret this statistic.

|  | Intelligence Attribution | Effort  Attribution | Fairness |
| --- | --- | --- | --- |
| Employee gender (ref. Man employee) |  |  |  |
| Woman employee | 0.697^***^ | 0.598^***^ | 0.933^***^ |
|  | (0.086) | (0.089) | (0.081) |
| Organization commits to... (ref. Uniform performance standards) |  |  |  |
| Women's advancement | 0.281^**^ | 0.076 | 0.242^**^ |
|  | (0.093) | (0.094) | (0.089) |
| Equal opportunities | 0.182^*^ | 0.036 | 0.255^**^ |
|  | (0.087) | (0.089) | (0.083) |
| Employee gender x organizational commitment |  |  |  |
| Woman employee x women's advancement | -0.523^***^ | -0.470^***^ | -0.484^***^ |
|  | (0.129) | (0.132) | (0.123) |
| Woman employee x equal opportunities | -0.332^**^ | -0.309^*^ | -0.355^**^ |
|  | (0.123) | (0.125) | (0.117) |
| Competitor gender (ref. Man competitor) |  |  |  |
| Woman competitor | -0.219^***^ | -0.158^**^ | -0.388^***^ |
|  | (0.051) | (0.052) | (0.049) |
| Resp. gender (ref. Woman respondent) |  |  |  |
| Man respondent | -0.002 | -0.009 | -0.039 |
|  | (0.052) | (0.053) | (0.050) |
| Non-binary respondent | -0.029 | 0.742 | 0.712^+^ |
|  | (0.244) | (0.474) | (0.405) |
| Age group in decades (ref. 20s) | 0.000 | 0.000 | 0.000 |
| 30s | -0.175 | -0.437^**^ | -0.381^**^ |
|  | (0.129) | (0.140) | (0.130) |
| 40s | -0.334^**^ | -0.443^**^ | -0.370^**^ |
|  | (0.127) | (0.135) | (0.125) |
| 50s | -0.280^*^ | -0.429^***^ | -0.450^***^ |
|  | (0.118) | (0.127) | (0.117) |
| 60s | -0.175 | -0.362^**^ | -0.396^***^ |
|  | (0.122) | (0.131) | (0.120) |
| Resp. relationship status (ref. Single, divorced, or widowed) |  |  |  |
| Married or in civil partnership | 0.045 | 0.034 | 0.060 |
|  | (0.058) | (0.059) | (0.056) |
| Resp. has children under age 18 in household (ref. No children) |  |  |  |
| Children under 18 in household | 0.014 | 0.036 | -0.023 |
|  | (0.068) | (0.071) | (0.068) |
| Resp. region of upbringing (ref. West Germany) |  |  |  |
| East Germany | 0.014 | -0.029 | -0.028 |
|  | (0.062) | (0.063) | (0.058) |
| Outside Germany | 0.206 | 0.302^+^ | 0.019 |
|  | (0.172) | (0.158) | (0.175) |
| Resp. education (ref. No tertiary degree) | 0.000 | 0.000 | 0.000 |
| Tertiary degree | -0.113^*^ | -0.157^**^ | -0.043 |
|  | (0.055) | (0.056) | (0.053) |
| Resp. subjective income assessment (ref. can live comfortably) |  |  |  |
| Can get by | 0.113^+^ | 0.113^+^ | 0.045 |
|  | (0.058) | (0.059) | (0.055) |
| Hard/very hard to get by | 0.077 | 0.015 | -0.001 |
|  | (0.092) | (0.091) | (0.089) |
| Resp. work status (ref. Working) |  |  |  |
| Not working | 0.093 | 0.133^+^ | 0.088 |
|  | (0.075) | (0.077) | (0.070) |
| Constant | 4.422^***^ | 4.969^***^ | 5.001^***^ |
|  | (0.134) | (0.144) | (0.134) |
| *N* | 3229 | 3229 | 3229 |

Table S4. Linear Regression Estimates for Effects of Organizational Commitments Interacted with Employee Gender. + p < 0.10, * p < 0.05, ** p < 0.01, *** p < 0.001; Two-tailed tests. Robust standard errors in parentheses. Only 5 respondents identified as non-binary, so we caution readers not to interpret this statistic.

| Organization commits to… | Intelligence Attribution | Effort  Attribution | Fairness Evaluation |
| --- | --- | --- | --- |
|  |  |  |  |
| Uniform performance standards | 0.697^***^ | 0.598^***^ | 0.933^***^ |
|  | (0.086) | (0.090) | (0.081) |
| Women’s advancement | 0.174^+^ | 0.127 | 0.448^***^ |
|  | (0.095) | (0.097) | (0.092) |
| Equal opportunities | 0.365^***^ | 0.288^***^ | 0.578^***^ |
|  | (0.087) | (0.087) | (0.084) |

Table S5. Contrasts of Marginal Linear Predictions of being a Woman compared to a Man Employee, by Organizational Commitments. + p < 0.10, * p < 0.05, ** p < 0.01, *** p < 0.001; Two-tailed tests. Robust standard errors in parentheses. N = 3,229. The predictions in this table were estimated based on the regression models in Table S4.

| Intelligence Attribution | | | | |
| --- | --- | --- | --- | --- |
|  | Women Employees | | Men Employees | |
|  | No controls | Initial sample | No controls | Initial sample |
| Organization commits to... (ref. Uniform performance standards) |  |  |  |  |
| Women's advancement | -0.245^**^ | -0.271^***^ | 0.271^**^ | 0.244^**^ |
|  | (0.089) | (0.078) | (0.093) | (0.080) |
| Equal opportunities | -0.157^+^ | -0.132^+^ | 0.179^*^ | 0.144^+^ |
|  | (0.086) | (0.074) | (0.087) | (0.078) |
| Competitor gender (ref. Man competitor) |  |  |  |  |
| Woman competitor | -0.134^+^ | -0.137^*^ | -0.299^***^ | -0.353^***^ |
|  | (0.072) | (0.062) | (0.073) | (0.065) |
| Resp. gender (ref. Woman respondent) |  |  |  |  |
| Man respondent |  | -0.227^***^ |  | 0.143^*^ |
|  |  | (0.063) |  | (0.065) |
| Non-binary respondent |  | -0.490 |  | 0.648 |
|  |  | (0.300) |  | (0.417) |
| Age group in decades (ref. 20s) |  |  |  |  |
| 30s |  | -0.373^*^ |  | -0.152 |
|  |  | (0.169) |  | (0.160) |
| 40s |  | -0.536^**^ |  | -0.086 |
|  |  | (0.164) |  | (0.157) |
| 50s |  | -0.427^*^ |  | -0.164 |
|  |  | (0.154) |  | (0.148) |
| 60s |  | -0.227 |  | -0.133 |
|  |  | (0.158) |  | (0.152) |
| Resp. relationship status (ref. Single, divorced, or widowed) |  |  |  |  |
| Married or in civil partnership |  | 0.059 |  | 0.005 |
|  |  | (0.071) |  | (0.071) |
| Resp. has children under age 18 in household (ref. No children) |  |  |  |  |
| Children under 18 in household |  | -0.001 |  | -0.027 |
|  |  | (0.085) |  | (0.082) |
| Resp. region of upbringing (ref. West Germany) |  |  |  |  |
| East Germany |  | 0.083 |  | -0.015 |
|  |  | (0.073) |  | (0.078) |
| Outside Germany |  | 0.051 |  | 0.302 |
|  |  | (0.220) |  | (0.197) |
| Resp. education (ref. No tertiary degree) |  |  |  |  |
| Tertiary degree |  | 0.044 |  | -0.213^**^ |
|  |  | (0.065) |  | (0.072) |
| Resp. subjective income assessment (ref. can live comfortably) |  |  |  |  |
| Can get by |  | 0.039 |  | 0.151^*^ |
|  |  | (0.071) |  | (0.075) |
| Hard/very hard to get by |  | -0.026 |  | 0.088 |
|  |  | (0.109) |  | (0.111) |
| Resp. work status (ref. Working) |  |  |  |  |
| Not working |  | 0.021 |  | 0.122 |
|  |  | (0.090) |  | (0.093) |
| Constant | 4.911^***^ | 5.345^***^ | 4.297^***^ | 4.457^***^ |
|  | (0.068) | (0.169) | (0.072) | (0.157) |
| *N* | 1613 | 2132 | 1616 | 2058 |

Table S6. Sensitivity Analyses: Linear Regression Estimates for Effects of Organizational Commitments on Intelligence Attributions, by Employee Gender. + p < 0.10, * p < 0.05, ** p < 0.01, *** p < 0.001; Two-tailed tests. Robust standard errors in parentheses. Only 5 respondents identified as non-binary, so we caution readers not to interpret this statistic.

|  | Effort Attribution | | | | |  |  |
| --- | --- | --- | --- | --- | --- | --- | --- |
|  | | | Women Employees | | Men Employees | | |
|  | | | No controls | Initial sample | No controls | Initial sample | |
| Organization commits to... (ref. Uniform performance standards) | | |  |  |  |  | |
|  | | |  |  |  |  | |
| Women's advancement | | | -0.400^***^ | -0.393^***^ | 0.070 | 0.064 | |
|  | | | (0.092) | (0.079) | (0.094) | (0.081) | |
| Equal opportunities | | | -0.285^**^ | -0.245^**^ | 0.033 | 0.020 | |
|  | | | (0.088) | (0.075) | (0.089) | (0.079) | |
| Competitor gender (ref. Man competitor) | | |  |  |  |  | |
|  | | |  |  |  |  | |
| Woman competitor | | | -0.017 | -0.036 | -0.293^***^ | -0.349^***^ | |
|  | | | (0.074) | (0.063) | (0.074) | (0.066) | |
| Resp. gender (ref. Woman respondent) | | |  |  |  |  | |
|  | | |  |  |  |  | |
| Man respondent | | |  | -0.238^***^ |  | 0.142^*^ | |
|  | | |  | (0.063) |  | (0.066) | |
| Non-binary respondent | | |  | -0.249 |  | 0.657 | |
|  | | |  | (0.460) |  | (0.786) | |
| Age group in decades (ref. 20s) | | |  |  |  |  | |
|  | | |  |  |  |  | |
| 30s | | |  | -0.480^**^ |  | -0.280 | |
|  | | |  | (0.173) |  | (0.179) | |
| 40s | | |  | -0.538^**^ |  | -0.225 | |
|  | | |  | (0.165) |  | (0.177) | |
| 50s | | |  | -0.511^**^ |  | -0.210 | |
|  | | |  | (0.156) |  | (0.168) | |
| 60s | | |  | -0.388^*^ |  | -0.207 | |
|  | | |  | (0.160) |  | (0.171) | |
| Resp. relationship status (ref. Single, divorced, or widowed) | | |  |  |  |  | |
|  | | |  |  |  |  | |
| Married or in civil partnership | | |  | 0.003 |  | 0.011 | |
|  | | |  | (0.072) |  | (0.072) | |
| Resp. has children under age 18 in household (ref. No children) | | |  |  |  |  | |
|  | | |  |  |  |  | |
| Children under 18 in household | | |  | 0.010 |  | 0.049 | |
|  | | |  | (0.087) |  | (0.086) | |
| Resp. region of upbringing (ref. West Germany) | | |  |  |  |  | |
|  | | |  |  |  |  | |
| East Germany | | |  | 0.041 |  | -0.068 | |
|  | | |  | (0.072) |  | (0.078) | |
| Outside Germany | | |  | -0.115 |  | 0.473^*^ | |
|  | | |  | (0.210) |  | (0.184) | |
| Resp. education (ref. No tertiary degree) | | |  |  |  |  | |
|  | | |  |  |  |  | |
| Tertiary degree | | |  | 0.005 |  | -0.269^***^ | |
|  | | |  | (0.067) |  | (0.072) | |
| Resp. subjective income assessment (ref. can live comfortably) | | |  |  |  |  | |
|  | | |  |  |  |  | |
| Can get by | | |  | 0.058 |  | 0.126^+^ | |
|  | | |  | (0.071) |  | (0.074) | |
| Hard/very hard to get by | | |  | -0.106 |  | -0.002 | |
|  | | |  | (0.108) |  | (0.111) | |
| Resp. work status (ref. Working) | | |  |  |  |  | |
|  | | |  |  |  |  | |
| Not working | | |  | 0.164^+^ |  | 0.142 | |
|  | | |  | (0.090) |  | (0.095) | |
| Constant | | | 5.140^***^ | 5.696^***^ | 4.678^***^ | 4.912^***^ | |
|  | | | (0.071) | (0.169) | (0.076) | (0.177) | |
| *N* | | | 1613 | 2132 | 1616 | 2058 | |

Table S7. Sensitivity Analyses: Linear Regression Estimates for Effects of Organizational Commitments on Effort Attributions, by Employee Gender. + p < 0.10, * p < 0.05, ** p < 0.01, *** p < 0.001; Two-tailed tests. Robust standard errors in parentheses. Only 5 respondents identified as non-binary, so we caution readers not to interpret this statistic.

|  | Fairness Evaluation | | | | |  |  |
| --- | --- | --- | --- | --- | --- | --- | --- |
|  | | | Women Employees | | Men Employees | | |
|  | | | No controls | Initial sample | No controls | Initial sample | |
| Organization commits to... (ref. Uniform performance standards) | | |  |  |  |  | |
|  | | |  |  |  |  | |
| Women's advancement | | | -0.241^**^ | -0.237^**^ | 0.238^**^ | 0.174^*^ | |
|  | | | (0.085) | (0.074) | (0.089) | (0.077) | |
| Equal opportunities | | | -0.101 | -0.094 | 0.243^**^ | 0.187^*^ | |
|  | | | (0.082) | (0.071) | (0.082) | (0.074) | |
| Competitor gender (ref. Man competitor) | | |  |  |  |  | |
|  | | |  |  |  |  | |
| Woman competitor | | | -0.122^+^ | -0.144^*^ | -0.655^***^ | -0.630^***^ | |
|  | | | (0.069) | (0.060) | (0.069) | (0.062) | |
| Resp. gender (ref. Woman respondent) | | |  |  |  |  | |
|  | | |  |  |  |  | |
| Man respondent | | |  | -0.305^***^ |  | 0.253^***^ | |
|  | | |  | (0.061) |  | (0.063) | |
| Non-binary respondent | | |  | 0.185 |  | 0.995^+^ | |
|  | | |  | (0.531) |  | (0.525) | |
| Age group in decades (ref. 20s) | | |  |  |  |  | |
|  | | |  |  |  |  | |
| 30s | | |  | -0.280^+^ |  | -0.438^**^ | |
|  | | |  | (0.165) |  | (0.160) | |
| 40s | | |  | -0.311^*^ |  | -0.509^**^ | |
|  | | |  | (0.157) |  | (0.155) | |
| 50s | | |  | -0.254^+^ |  | -0.622^***^ | |
|  | | |  | (0.151) |  | (0.145) | |
| 60s | | |  | -0.142 |  | -0.587^***^ | |
|  | | |  | (0.154) |  | (0.147) | |
| Resp. relationship status (ref. Single, divorced, or widowed) | | |  |  |  |  | |
|  | | |  |  |  |  | |
| Married or in civil partnership | | |  | -0.110 |  | 0.163^*^ | |
|  | | |  | (0.069) |  | (0.068) | |
| Resp. has children under age 18 in household (ref. No children) | | |  |  |  |  | |
|  | | |  |  |  |  | |
| Children under 18 in household | | |  | 0.056 |  | -0.026 | |
|  | | |  | (0.082) |  | (0.084) | |
| Resp. region of upbringing (ref. West Germany) | | |  |  |  |  | |
|  | | |  |  |  |  | |
| East Germany | | |  | 0.013 |  | -0.040 | |
|  | | |  | (0.069) |  | (0.071) | |
| Outside Germany | | |  | -0.190 |  | 0.192 | |
|  | | |  | (0.193) |  | (0.225) | |
| Resp. education (ref. No tertiary degree) | | |  |  |  |  | |
|  | | |  |  |  |  | |
| Tertiary degree | | |  | 0.036 |  | -0.137^*^ | |
|  | | |  | (0.064) |  | (0.068) | |
| Resp. subjective income assessment (ref. can live comfortably) | | |  |  |  |  | |
|  | | |  |  |  |  | |
| Can get by | | |  | 0.115^+^ |  | -0.043 | |
|  | | |  | (0.068) |  | (0.070) | |
| Hard/very hard to get by | | |  | -0.035 |  | -0.095 | |
|  | | |  | (0.104) |  | (0.108) | |
| Resp. work status (ref. Working) | | |  |  |  |  | |
|  | | |  |  |  |  | |
| Not working | | |  | 0.121 |  | 0.081 | |
|  | | |  | (0.083) |  | (0.088) | |
| Constant | | | 5.433^***^ | 5.780^***^ | 4.783^***^ | 5.241^***^ | |
|  | | | (0.066) | (0.164) | (0.067) | (0.156) | |
| *N* | | | 1613 | 2132 | 1616 | 2058 | |

Table S8. Sensitivity Analyses: Linear Regression Estimates for Effects of Organizational Commitments on Fairness Evaluations by Employee Gender. + p < 0.10, * p < 0.05, ** p < 0.01, *** p < 0.001; Two-tailed tests. Robust standard errors in parentheses. Only 5 respondents identified as non-binary, so we caution readers not to interpret this statistic.

|  | | Intelligence Attribution | | | | Effort Attribution | | | Fairness Attribution | | |
| --- | --- | --- | --- | --- | --- | --- | --- | --- | --- | --- | --- |
|  | Women Employees | | Men  Employees | | Women  Employees | | | Men  Employees | Women  Employees | | Men  Employees |
| Organization commits to... (ref. Uniform performance standards) |  | |  | |  | | |  |  | |  |
|  |  | |  | |  | | |  |  | |  |
| Women's advancement | -0.293^**^ | | 0.348^**^ | | -0.482^***^ | | | 0.102 | -0.279^*^ | | 0.277^*^ |
|  | (0.109) | | (0.111) | | (0.110) | | | (0.110) | (0.109) | | (0.112) |
| Equal opportunities | -0.187^+^ | | 0.221^*^ | | -0.363^***^ | | | 0.014 | -0.130 | | 0.278^**^ |
|  | (0.108) | | (0.107) | | (0.107) | | | (0.107) | (0.109) | | (0.108) |
| Competitor gender (ref. Man competitor) |  | |  | |  | | |  |  | |  |
|  |  | |  | |  | | |  |  | |  |
| Woman competitor | -0.188^*^ | | -0.348^***^ | | -0.064 | | | -0.337^***^ | -0.204^*^ | | -0.857^***^ |
|  | (0.089) | | (0.090) | | (0.088) | | | (0.089) | (0.089) | | (0.092) |
| Resp. gender (ref. Woman respondent) |  | |  | |  | | |  |  | |  |
|  |  | |  | |  | | |  |  | |  |
| Man respondent | -0.321^***^ | | 0.288^**^ | | -0.330^***^ | | | 0.272^**^ | -0.492^***^ | | 0.336^***^ |
|  | (0.090) | | (0.090) | | (0.089) | | | (0.090) | (0.090) | | (0.091) |
| Non-binary respondent | -0.657 | | 0.334 | | -0.553 | | | 1.579 | 0.962 | | 0.740 |
|  | (1.106) | | (0.896) | | (1.056) | | | (1.031) | (1.259) | | (0.957) |
| Age group in decades (ref. 20s) |  | |  | |  | | |  |  | |  |
|  |  | |  | |  | | |  |  | |  |
| 30s | -0.288 | | -0.185 | | -0.677^**^ | | | -0.452^+^ | -0.470^+^ | | -0.532^*^ |
|  | (0.254) | | (0.239) | | (0.261) | | | (0.246) | (0.260) | | (0.246) |
| 40s | -0.613^*^ | | -0.263 | | -0.767^**^ | | | -0.399^+^ | -0.414^+^ | | -0.568^*^ |
|  | (0.244) | | (0.233) | | (0.250) | | | (0.239) | (0.249) | | (0.238) |
| 50s | -0.487^*^ | | -0.235 | | -0.742^**^ | | | -0.363 | -0.316 | | -0.824^***^ |
|  | (0.229) | | (0.220) | | (0.235) | | | (0.226) | (0.234) | | (0.223) |
| 60s | -0.287 | | -0.226 | | -0.610^*^ | | | -0.369 | -0.237 | | -0.763^***^ |
|  | (0.233) | | (0.224) | | (0.240) | | | (0.230) | (0.239) | | (0.227) |
| Resp. relationship status (ref. Single, divorced, or widowed) |  | |  | |  | | |  |  | |  |
|  |  | |  | |  | | |  |  | |  |
| Married or in civil partnership | 0.109 | | 0.015 | | 0.060 | | | 0.048 | -0.080 | | 0.250^*^ |
|  | (0.101) | | (0.097) | | (0.100) | | | (0.097) | (0.102) | | (0.098) |
| Resp. has children under age 18 in household (ref. No children) |  | |  | |  | | |  |  | |  |
|  |  | |  | |  | | |  |  | |  |
| Children under 18 in household | 0.061 | | -0.014 | | 0.056 | | | 0.049 | 0.056 | | -0.127 |
|  | (0.123) | | (0.119) | | (0.123) | | | (0.119) | (0.123) | | (0.123) |
| Resp. region of upbringing (ref. West Germany) |  | |  | |  | | |  |  | |  |
|  |  | |  | |  | | |  |  | |  |
| East Germany | 0.137 | | -0.111 | | 0.042 | | | -0.099 | 0.073 | | -0.097 |
|  | (0.104) | | (0.106) | | (0.104) | | | (0.106) | (0.105) | | (0.105) |
| Outside Germany | 0.142 | | 0.360 | | 0.162 | | | 0.593^+^ | -0.190 | | 0.362 |
|  | (0.280) | | (0.314) | | (0.278) | | | (0.304) | (0.277) | | (0.340) |
| Resp. education (ref. No tertiary degree) |  | |  | |  | | |  |  | |  |
|  |  | |  | |  | | |  |  | |  |
| Tertiary degree | 0.008 | | -0.285^**^ | | 0.002 | | | -0.393^***^ | 0.034 | | -0.207^*^ |
|  | (0.094) | | (0.097) | | (0.093) | | | (0.097) | (0.095) | | (0.098) |
| Resp. subjective income assessment (ref. can live comfortably) |  | |  | |  | | |  |  | |  |
|  |  | |  | |  | | |  |  | |  |
| Can get by | 0.024 | | 0.221^*^ | | 0.102 | | | 0.215^*^ | 0.173^+^ | | -0.080 |
|  | (0.100) | | (0.101) | | (0.100) | | | (0.100) | (0.100) | | (0.101) |
| Hard/very hard to get by | 0.015 | | 0.214 | | 0.046 | | | 0.009 | 0.042 | | -0.047 |
|  | (0.153) | | (0.154) | | (0.152) | | | (0.152) | (0.155) | | (0.155) |
| Resp. work status (ref. Working) |  | |  | |  | | |  |  | |  |
|  |  | |  | |  | | |  |  | |  |
| Not working | 0.048 | | 0.251^+^ | | 0.211^+^ | | | 0.207 | 0.138 | | 0.057 |
|  | (0.126) | | (0.129) | | (0.126) | | | (0.129) | (0.127) | | (0.130) |
|  |  | |  | |  | | |  |  | |  |
| Cut1 | -4.191^***^ | | | -3.044^***^ | -4.705^***^ | | -3.708^***^ | | -5.275^***^ | -4.550^***^ | |
|  | (0.287) | | | (0.258) | (0.299) | | (0.275) | | (0.351) | (0.287) | |
| Cut2 | -3.224^***^ | | | -2.112^***^ | -3.615^***^ | | -2.684^***^ | | -4.194^***^ | -3.478^***^ | |
|  | (0.264) | | | (0.245) | (0.270) | | (0.255) | | (0.291) | (0.261) | |
| Cut3 | -2.265^***^ | | | -1.135^***^ | -2.658^***^ | | -1.713^***^ | | -3.231^***^ | -2.438^***^ | |
|  | (0.255) | | | (0.239) | (0.260) | | (0.248) | | (0.269) | (0.250) | |
| Cut4 | -0.951^***^ | | | 0.200 | -1.517^***^ | | -0.516^*^ | | -1.384^***^ | -0.692^**^ | |
|  | (0.249) | | | (0.236) | (0.255) | | (0.244) | | (0.256) | (0.242) | |
| Cut5 | 0.166 | | | 1.348^***^ | -0.492^+^ | | 0.605^*^ | | -0.540^*^ | 0.210 | |
|  | (0.248) | | | (0.239) | (0.253) | | (0.244) | | (0.254) | (0.242) | |
| Cut6 | 1.336^***^ | | | 2.504^***^ | 0.706^**^ | | 1.763^***^ | | 0.434^+^ | 1.238^***^ | |
|  | (0.253) | | | (0.250) | (0.253) | | (0.250) | | (0.254) | (0.246) | |
|  |  | |  | |  | | |  |  | |  |
| *N* | 1613 | | 1616 | | 1613 | | | 1616 | 1613 | | 1616 |

Table S9. Sensitivity Analyses: Ordered Logistic Regression Estimates for Effects of Organizational Commitments by Employee Gender. Ordered logistic regression coefficients. + p < 0.10, * p < 0.05, ** p < 0.01, *** p < 0.001; Two-tailed tests. Standard errors in parentheses. Only 5 respondents identified as non-binary, so we caution readers not to interpret this statistic.

|  | Intelligence Attribution | | Effort Attribution | | Fairness | |
| --- | --- | --- | --- | --- | --- | --- |
|  | Women Employees | Men Employees | Women Employees | Men Employees | Women Employees | Men Employees |
| Organization commits to... (ref. Uniform performance standards) |  |  |  |  |  |  |
| Women's advancement | -0.388^**^ | 0.327^**^ | -0.768^***^ | 0.111 | -0.490^**^ | 0.271^**^ |
|  | (7.86) | (9.56) | (19.29) | (0.77) | (7.51) | (8.52) |
| Equal Opportunities | -0.250^+^ | 0.209^*^ | -0.579^***^ | 0.037 | -0.214 | 0.277^**^ |
|  | (3.26) | (4.14) | (10.97) | (0.09) | (1.43) | (9.34) |
| Competitor gender (ref. Man competitor) |  |  |  |  |  |  |
| Woman Competitor | -0.232^*^ | -0.347^***^ | -0.080 | -0.393^***^ | -0.318^*^ | -0.693^***^ |
|  | (4.21) | (16.30) | (0.31) | (14.76) | (4.71) | (82.34) |
| Resp. gender (ref. Woman respondent) |  |  |  |  |  |  |
| Man Respondent | -0.418^***^ | 0.289^***^ | -0.539^***^ | 0.331^**^ | -0.799^***^ | 0.320^***^ |
|  | (13.37) | (11.15) | (13.95) | (10.27) | (29.08) | (17.69) |
| Non-binary Respondent | -0.876 | 0.321 | -0.893 | 1.993^+^ | 1.771 | 0.687 |
|  | (0.29) | (0.11) | (0.19) | (2.87) | (0.72) | (0.63) |
| Age group in decades (ref. 20s) |  |  |  |  |  |  |
| 30s | -0.373 | -0.151 | -1.126^**^ | -0.466^+^ | -0.734^+^ | -0.467^*^ |
|  | (1.26) | (0.40) | (7.22) | (2.71) | (2.93) | (4.99) |
| 40s | -0.821^**^ | -0.206 | -1.264^**^ | -0.414 | -0.686^+^ | -0.482^*^ |
|  | (6.74) | (0.80) | (10.01) | (2.25) | (2.82) | (5.63) |
| 50s | -0.631^*^ | -0.209 | -1.226^**^ | -0.394 | -0.561 | -0.693^***^ |
|  | (4.51) | (0.93) | (10.67) | (2.32) | (2.13) | (13.17) |
| 60s | -0.356 | -0.203 | -0.983^**^ | -0.397 | -0.404 | -0.661^***^ |
|  | (1.38) | (0.85) | (6.58) | (2.29) | (1.06) | (11.68) |
| Resp. relationship status (ref. Single, divorced, or widowed) |  |  |  |  |  |  |
| Married or civil partnership | 0.124 | 0.009 | 0.087 | 0.038 | -0.123 | 0.201^*^ |
|  | (0.94) | (0.01) | (0.29) | (0.11) | (0.55) | (5.97) |
| Resp. has children under age 18 in household (ref. No children) |  |  |  |  |  |  |
| Children under 18 in household | 0.064 | -0.031 | 0.109 | 0.026 | 0.066 | -0.080 |
|  | (0.16) | (0.07) | (0.30) | (0.04) | (0.11) | (0.62) |
| Resp. region of upbringing (ref. West Germany) |  |  |  |  |  |  |
| East Germany | 0.188 | -0.062 | 0.094 | -0.094 | 0.101 | -0.073 |
|  | (2.00) | (0.39) | (0.31) | (0.63) | (0.35) | (0.70) |
| Outside Germany | 0.188 | 0.423 | 0.292 | 0.736^*^ | -0.333 | 0.282 |
|  | (0.29) | (2.00) | (0.44) | (4.25) | (0.54) | (1.15) |
| Resp. education (ref. No tertiary degree) |  |  |  |  |  |  |
| Tertiary degree | -0.001 | -0.281^**^ | -0.004 | -0.464^***^ | 0.058 | -0.163 |
|  | (0.00) | (9.26) | (0.00) | (17.73) | (0.14) | (4.03) |
| Resp. subjective income assessment (ref. can live comfortably) |  |  |  |  |  |  |
| Can get by | 0.044 | 0.218^*^ | 0.120 | 0.234^*^ | 0.287^+^ | -0.057 |
|  | (0.12) | (5.12) | (0.56) | (4.17) | (3.01) | (0.45) |
| Hard/very hard to get by | -0.008 | 0.214 | 0.028 | 0.038 | 0.066 | -0.033 |
|  | (0.00) | (2.17) | (0.01) | (0.05) | (0.07) | (0.07) |
| Resp. work status (ref. Working) |  |  |  |  |  |  |
| Not working | 0.030 | 0.250^*^ | 0.333^+^ | 0.189 | 0.226 | 0.076 |
|  | (0.03) | (4.15) | (2.72) | (1.68) | (1.20) | (0.49) |
| Constant | 6.085 | 3.721 | 8.032 | 4.940 | 8.544 | 4.419 |
|  |  |  |  |  |  |  |
| Theta |  |  |  |  |  |  |
| Constant | 1.312 | 1.114 | 1.444 | 1.223 | 1.478 | 1.050 |
|  |  |  |  |  |  |  |
| Sigma |  |  |  |  |  |  |
| Constant | 2.265 | 1.701 | 2.858 | 2.028 | 2.928 | 1.495 |
|  |  |  |  |  |  |  |
| *N* | 1613 | 1616 | 1613 | 1616 | 1613 | 1616 |

Table S10. Sensitivity Analyses: Box-Cox Transformed Dependent Variable Regression Analyses for Effects of Organizational Commitments by Employee Gender.: LR statistics for tests on Independent Variables in Parentheses. P-values for LR tests on Independent Variables: + p < 0.10, * p < 0.05, ** p < 0.01, *** p < 0.001. Only 5 respondents identified as non-binary, so we caution readers not to interpret this statistic.
